# The Surgical Renaissance: Advancements in Video-Assisted Thoracoscopic Surgery and Robotic-Assisted Thoracic Surgery and Their Impact on Patient Outcomes

**DOI:** 10.3390/cancers16173086

**Published:** 2024-09-05

**Authors:** Jennifer M. Pan, Ammara A. Watkins, Cameron T. Stock, Susan D. Moffatt-Bruce, Elliot L. Servais

**Affiliations:** 1 Division of General Surgery, Beth Israel Deaconess Medical Center, Boston, MA 02215, USA; 2 Division of Cardiothoracic Surgery, Lahey Hospital and Medical Center, Burlington, MA 01805, USA

**Keywords:** VATS, lung cancer, RATS, minimally invasive surgery, thoracic outcomes

## Abstract

**Simple Summary:**

Lung cancer treatment often involves surgical resection. Traditional resection involves a large thoracotomy incision to access the chest cavity. However, thoracotomy has become less common since the introduction of minimally invasive surgery (MIS). Minimally invasive surgery includes video-assisted thoracoscopic surgery (VATS) and robotic-assisted thoracic surgery (RATS) and involves small incisions to accomplish the same operation. MIS techniques have improved patient outcomes compared to thoracotomy. Patients recover faster in the hospital, have fewer complications and less pain, and return to baseline function faster. These techniques have now become the standard of care for lung cancer resection.

**Abstract:**

Minimally invasive thoracic surgery has advanced the treatment of lung cancer since its introduction in the 1990s. Video-assisted thoracoscopic surgery (VATS) and robotic-assisted thoracic surgery (RATS) offer the advantage of smaller incisions without compromising patient outcomes. These techniques have been shown to be safe and effective in standard pulmonary resections (lobectomy and sub-lobar resection) and in complex pulmonary resections (sleeve resection and pneumonectomy). Furthermore, several studies show these techniques enhance patient outcomes from early recovery to improved quality of life (QoL) and excellent oncologic results. The rise of RATS has yielded further operative benefits compared to thoracoscopic surgery. The wristed instruments, neutralization of tremor, dexterity, and magnification allow for more precise and delicate dissection of tissues and vessels. This review summarizes of the advancements in minimally invasive thoracic surgery and the positive impact on patient outcomes.

## 1. Introduction of Minimally Invasive Lung Resection

Surgery remains a cornerstone therapy in lung cancer. Pulmonary resection is the mainstay treatment for early-stage lung cancer [1]. As the pharmacologic treatment of lung cancer improves, surgery in locally advanced-stage lung cancer has become more feasible [2,3,4]. With this, there have been dramatic advancements in operative techniques and modalities for lung resection. As minimally invasive surgery (MIS) has grown in popularity in other fields, it has also been eagerly adopted by thoracic surgeons. MIS was first introduced in the 1990s. The first video-assisted thoracoscopic surgery (VATS) cases were described in the literature over thirty years ago [5]. Since then, multiple studies have examined and established the feasibility, safety, and advantages of VATS. In the early 2000s, robotic-assisted thoracoscopic surgery (RATS) was introduced. The first case series demonstrated the flexibility of the robotic platform to perform lobectomies, tumor resections, enucleations, and even bulla stitching [6]. 

**Figure 1:**
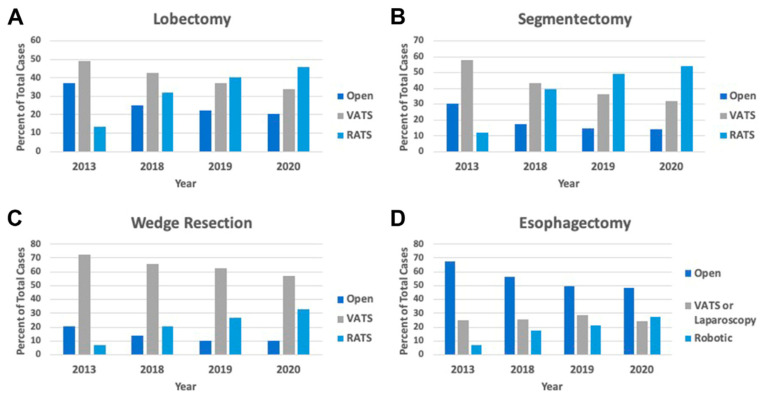
Distribution of cases and modalities over time. Modalities were thoracotomy (dark blue bars), VATS (grey bars), and RATS (blue bars). ([2022] Servais et al. Reproduced with permission from the authors [9]).

The field of thoracic surgery has transformed since the introduction of minimally invasive surgery. Open thoracotomy was previously regarded as the gold standard in lung cancer treatment [7]. Over the past few decades, VATS and robotic thoracic surgery have consistently demonstrated improved patient outcomes, from fewer postoperative complications to shorter hospital stays and appropriate oncologic safety [8]. The improvements in patient outcomes have resulted in a change in the standard of care. The adoption of minimally invasive surgery has increased tremendously over the past decade [9,10]. Minimally invasive resections have become the standard of care in lung cancer treatment [8]. In fact, according to a 2023 review of the Society of Thoracic Surgeons General Thoracic Surgery Database (STS-GTSD), over 80% of lobectomies, segmentectomies, and wedge resections were performed via a minimally invasive approach (Figure 1) [9]. 

The objective of this review is to summarize the advancements in minimally invasive techniques for lung cancer and the impact on patient outcomes.

## 2. Video-Assisted Thoracoscopic Surgery for Lung Cancer

Since the introduction of VATS, numerous studies have demonstrated either equivalent or improved outcomes compared to thoracotomy. VATS has been shown to be noninferior or better for patients in several realms, particularly in early-stage lung cancer. The CALGB309802 study was one of the first prospective studies to examine the outcomes of patients who underwent a VATS approach. They established that VATS lobectomy outcomes were comparable to those after a thoracotomy [11]. Multiple studies have found no significant difference in operative time [12,13,14,15]. The majority of comparative studies of open and VATS lobectomy for early-stage lung cancer reported significantly lower intraoperative blood loss during VATS [16]. Yan et al. also reviewed median chest tube duration by approach. While chest tube duration was lower after VATS lobectomy, they were unable to draw statistical significance [16]. In another meta-analysis, Whitson et al. concluded that chest drain duration was significantly shorter after VATS than thoracotomy. In other postoperative outcomes, VATS has been established to be as safe as open thoracotomy [12]. The VIOLET trial, a recent randomized controlled trial (RCT), investigated postoperative outcomes in patients who underwent VATS or open lobectomies. There was no difference in serious adverse events between the two groups. While the VATS group had a higher rate of prolonged air leaks and bleeding, they had a shorter hospital course, a lower readmission rate, and less overall pain [15]. In another RCT, Long et al. randomized patients with early NSCLC to undergo lobectomy via VATS or thoracotomy (Clinicaltrials.gov NCT01102517). The operative time was significantly shorter, and blood loss was significantly lower after VATS. There was no difference in postoperative complications [17]. Multiple observational studies have found either an equivalent or lower rate of prolonged air leaks and other postoperative complications after VATS surgery [12,14,16,18,19,20]. Patients who underwent VATS lobectomy in the VIOLET trial had a shorter length of hospital stay, which is consistent with prior existing studies and meta-analyses [12,13,14,15,18,21,22]. 

VATS lobectomy for lung cancer has demonstrated equivalent or improved oncologic and survival outcomes compared to open lobectomy [13,14,16,18,20,21,23]. The recurrence rates of VATS are comparable to those of open thoracotomy [15,24]. In a meta-analysis, locoregional recurrence was similar between the two, but systemic recurrence was significantly lower for VATS [16]. The disease-free and overall survival rate between VATS and open is comparable [11,12,15,18,24]. The quality of lymph node dissection via VATS has been a much-debated topic. In a landmark trial, Z0030, no statistical differences were seen in the completeness of lymphadenectomy between VATS and thoracotomy approaches [25]. In fact, several studies have shown that an appropriate lymphadenectomy can be performed via a VATS approach for both total lymph node harvest and lymph node station sampling [14,15,18,19,24]. VATS is associated with a lower rate of upstaging by pathologic stage [26,27]. Patients who undergo VATS have been found to undergo more of their planned chemotherapy regimen with less of a need for reduced doses [28]. While there is a lower rate of upstaging, VATS has actually demonstrated either equivalent or improved survival compared to thoracotomy [26,27,29]. 

With modern advances in neoadjuvant treatment, more locally advanced and even late-stage lung cancer patients are now able to undergo oncologic lung resection. While there are no randomized control trials comparing VATS to open in these patients, several studies have shown similar outcomes between VATS and thoracotomy following induction therapy. In a retrospective review of patients who underwent neoadjuvant chemotherapy, VATS patients were sicker at baseline, with higher clinical stage, age, coronary disease, and chronic pulmonary disease. Despite having more comorbidities, VATS had improved 3-year survival compared to open [30]. Disease-free survival and recurrence rates were not significantly different [30,31,32]. In a review of the National Cancer Database (NCDB) of later-stage NSCLC patients, VATS and open had comparable resection outcomes, lymphadenectomy, and complication rates and similar overall survival. Patients after VATS had a shorter chest tube duration and length of stay [31]. Patients who underwent induction therapy did have a higher rate of conversion to open, with reasons being fibrotic tissue or nodes, adhesions, bleeding, or extensive involvement of the vasculature [4,33].

The advantages of VATS led to a shift in the 2000s, with increased use of VATS for lung cancer treatment. It became the new standard of care, particularly for early-stage NSCLC, with clear evidence showing similar or improved patient outcomes, from perioperative recovery to oncologic outcomes [34,35]. 

## 3. Robotic-Assisted Thoracic Surgery for Lung Cancer

Since the introduction of robotic surgery, RATS has become the most common approach for anatomic lung resections [9]. There are many advantages to the robotic platform compared to open and VATS. Minimally invasive approaches allow for dexterity and magnification. Compared to VATS, the robotic platform enhances maneuverability with wristed instruments, eliminates physiologic tremor, and provides three-dimensional visualization of the field. The robotic platform also routinely utilizes insufflation, which also improves visualization [36].

### 3.1. Robotic and Open Thoracotomy Outcomes

Robotic-assisted lung resection confers improved outcomes compared to thoracotomy [37,38,39,40,41]. In a large database study of the State Inpatient Database, Kent et al. compared the outcomes of thoracotomy compared to minimally invasive techniques. Patients who underwent robotic-assisted resection had significantly lower mortality, length of stay, and overall complication rates compared to thoracotomy [42]. In an analysis of the Premier Healthcare Database, the robotic approach was associated with longer operative times (275.5 vs. 235.3 min, *p* < 0.0001) but significantly fewer postoperative complications, shorter hospital stay, and higher rates of discharge home. Patients after RATS had less postoperative bleeding and fewer respiratory complications, including pneumonia and the need for mechanical ventilation [43]. These trends were also demonstrated by Nguyen et al., who compared open and robotic cases between 2013 and 2015 [44]. A meta-analysis of minimally invasive and open lobectomies found the same benefits of robotic-assisted surgery. Robotic-assisted lobectomy had a significant reduction in blood loss, chest tube duration, 30-day mortality, and overall complication rates, particularly pulmonary complications [45]. 

The improved outcomes are consistent in even later-stage disease. Zirafa et al. compared the post-surgical outcomes of locally advanced NSCLC. The operative time for robotic-assisted surgery was longer than open thoracotomy. Notably, the robot operative time did decrease over time and with the introduction of the da Vinci Xi system (Intuitive Surgical, Sunnyvale, CA, USA). Reasons may be due to improved surgeon and staff experience. The length of hospital stay and postoperative complication rates were lower in the robotic-assisted group [46]. In another analysis of patients with clinical T4 disease within the National Cancer Database, patients who underwent MIS resection had a shorter hospital stay. There were no differences in short-term mortality or overall survival [47].

Compared to open lobectomy, RATS lobectomy shows improvement in postoperative outcomes. Robotic resection is effective even for later-stage lung cancers, with similar outcomes to open approaches. 

### 3.2. RATS and VATS Outcomes

RATS has similar or improved outcomes to VATS surgery [43,48,49,50]. Agzarian et al. performed a systematic review of the two approaches. While the operative time for VATS was shorter than for RATS, there were no differences in perioperative outcomes, such as blood loss, prolonged air leak, chest tube duration, and length of stay [51]. In a more recent multi-center analysis of lobectomy cases, the operative time of a RATS lobectomy was significantly shorter than for VATS or open lobectomy. Additionally, patients who underwent a robotic-assisted surgery had lower transfusion rates and shorter hospital stays compared to those who underwent VATS [41]. The shorter robotic operative time was attributed to increased familiarity and experience with robotic-assisted surgery by the operative staff. 

Louie et al. found that patients who underwent robotic-assisted lobectomies had more comorbidities than those who underwent VATS. Patients who underwent robotic-assisted lung resection were more likely to be older and have a lower performance status, cardiac disease, and higher body mass index (BMI) [52]. Despite these differences, surgical outcomes were comparable, suggesting that robotic-assisted lobectomy may be preferable in older patients with comorbidities. A randomized controlled trial comparing RATS and VATS (ROMAN) found no difference in the duration of surgery, perioperative complications, or postoperative stay. The robotic approach yielded a more comprehensive sampling of lymph nodes [53]. In a more recent meta-analysis comparing minimally invasive pulmonary resections, perioperative morbidity was similar, but 30-day mortality was significantly lower after a robotic lobectomy or segmentectomy. They also identified a significantly lower conversion to open rate with the robotic approach [54]. Servais et al. analyzed the STS-GTSD for rates of conversion to thoracotomy (Table 1). RATS had a significantly lower rate of conversion compared to VATS (6.0% vs. 11.0%, *p* < 0.001). While conversion to open from RATS occurred more emergently and for vascular reasons, there was no difference in mortality [55]. Robotic-assisted lobectomy yields comparable, if not improved, intraoperative and postoperative outcomes compared to VATS.

## 4. Oncologic Outcomes

The quality of lymph node sampling and dissection has been shown to impact survival in patients with NSCLC [25,56,57]. The extent of lymph node sampling is an important operative quality metric. Oncologic impact after surgery is a vital measure of patient outcomes. The quality of minimally invasive lymph node evaluation has been reviewed extensively. Robot-assisted dissection results in improved nodal harvest compared to thoracotomy [38,58,59,60]. Wilson et al. conducted a multi-institutional review of early-stage NSCLC patients who underwent resection. The rate of overall robotic nodal upstaging was 10.9%. When separated by T stage, the rate of upstaging was comparable to that of thoracotomy and superior to VATS [58]. Higher rates of nodal harvest were also seen in a retrospective analysis of open, VATS, and RATS cases. Robotic nodal dissection yielded more overall nodes and more hilar lymph nodes compared to open and VATS [59]. A large retrospective review of stage I NSCLC found that more lymph node stations were dissected with the robotic approach than the open approach or VATS. While upstaging rates were not examined, the robotic group had a higher proportion of pathological N1 nodes [60]. The ROMAN RCT of early NSCLC found that the robotic approach was superior in dissection of both hilar and mediastinal lymph nodes compared to VATS in early-stage disease [53]. The improved lymph node outcomes with the robotic approach may be a result of improved visualization of the field and articulation of the instruments to allow for specific and delicate dissection.

Robotic-assisted surgery for lung cancer has equivalent overall survival and disease-free outcomes to open and VATS approaches [60,61,62,63]. Park et al. reported in a multi-center study of robotic lobectomies for stage I-III NSCLC an overall 1-year and 5-year survival rate of 98% and 80%, respectively, with comparable stage-specific survival to prior VATS data [64]. When comparing survival and recurrence outcomes for open, VATS, and RATS lobectomies for stage I-IIIa, there were no statistically significant differences between the three approaches [65]. These results were also demonstrated in a meta-analysis comparing the three modalities [45]. In patients with cT4 NSCLC, survival up to 84 months was similar between MIS and open resection. At five years, the survival rate after MIS was 53.4%, and after open, it was 51.2%, but this was not statistically significant [47]. Zirafa et al. compared outcomes after open and robotic resection for locally advanced lung cancer. They found that the overall 5-year survival was 34.3% and 31.0% for the robotic and open group, respectively. Progression of disease was found in 65.6% of patients and 68.6% of patients treated with robotic and open surgery, respectively. These differences were not statistically significant [46]. Between the MIS techniques, a recent meta-analysis identified similar overall survival; however, patients treated with robotic resection had a longer disease-free survival [66].

## 5. Quality of Life

Thoracotomy incisions are painful, and they can impede patient recovery. Patients who undergo a minimally invasive approach consistently have short hospital stays and more discharges to home [67]. The use of a rib spreader during VATS surgery leads to more pain medication use with slower recovery [68]. Early analysis of VATS and thoracotomy outcomes demonstrated that patients who underwent VATS had more independence at time of hospital discharge, less pain, faster return to work, and higher rates of initiating adjuvant chemotherapy [67]. The VIOLET trial found that patients who were randomized to VATS had improved physical function at five weeks compared to open thoracotomy. The thoracotomy group took an average of six months to achieve the same level of physical fitness that the VATS group achieved at five weeks after resection. Patients who underwent VATS also had less overall pain by postoperative day two. VATS patients continued to report lower pain scores up to a year after their operation [15]. In a randomized controlled trial, patients undergoing lobectomy for stage I NSCLC were randomly assigned to VATS or anterolateral thoracotomy (Clinicaltrials.gov NCT01278888). Patients who underwent VATS had significantly lower pain scores within 24 h of surgery. The difference in pain levels was sustained throughout the year after surgery. Quality of life (QoL) and emotional function were significantly improved in patients who were treated with VATS [22]. 

Cerfolio et al. analyzed his initial cohort of robotic resections and found significantly improved mental and physical QoL scores and lower pain scores at 3-week follow-up compared to thoracotomy [69]. Quality of life questionnaires were given to patients undergoing robotic, VATS, and open pulmonary resection. Patients treated with RATS resection had the highest measures of functional QoL and the fewest number of symptoms [70]. Lacroix et al. analyzed patients who underwent robotic or open lobectomy. The patients who underwent robotic lobectomy were given a weaker postoperative pain control protocol. They found no difference in pain between the two cohorts. At one month, patients who underwent RATS lobectomy had less pain and improved pulmonary function tests compared to the open group [71]. In a review of the SEER database of opioid use, open resection had increased persistent opioid use after surgery compared to RATS and VATS. Interestingly, this was only observed in opioid-naive patients. There was no difference in persistent opioid use in chronic-opioid-use patients [72]. Kwon et al. reviewed pain scores in 498 patients who underwent pulmonary resection. There was a significant improvement in acute pain in patients who underwent minimally invasive surgery compared to open surgery. VATS and RATS lung resections resulted in less pain, faster recovery, and improved QoL [73].

## 6. Cost

The cost of robotic surgery has been a concern, especially considering the upfront costs and system maintenance of a robotic system [74,75,76]. In 2006, Park et al. cited an initial purchase cost of USD 1 million with an annual maintenance cost of USD 100,000 and a technical cost of USD 730 on average per case [74]. In a cost comparison of open, VATS, and robotic surgery for lung cancer, the costs of open compared to robotic cases were not statistically significant. However, robotic cases on average were USD 3182 more than VATS (*p* < 0.001). A reduction of robotic operating time by 68 min or a reduction of a hospital stay by 1.86 days would eliminate the statistically significant difference in cost between VATS and RATS cases [77].

The cost trends of robotic-assisted lung resection have changed over time as more centers now own robotic systems and are performing robotic-assisted cases. In a review of the Premier database from 2009 to 2011, average robotic-assisted operation costs were significantly more expensive than VATS without any significant differences in length of stay or adverse events [78]. However, in a review of the Premier Healthcare Database from 2008 to 2015, the costs were more comparable. The authors divided the review period into an early (2008–2012) and late period (2013–2015). Hospitals with an annual case volume of >25 lobectomies during the late period had comparable costs among RATS, VATS, and open lobectomies. Additionally, during the late period, robotic-assisted lobectomies had significantly improved outcomes and shorter length of stay [44]. With the increase in robotic cases and training programs, familiarity and experience in performing robotic-assisted lung resections have improved. Kneuertz et al. reviewed their cost data from 2012 to 2017. Robotic operative times were significantly shorter than VATS and equivalent to open times. Overall costs for VATS and robotic-assisted surgery were comparable. The initial operative costs of open surgery were significantly lower than those of RATS, but those costs were recuperated in the postoperative period [79]. Alwatari et al. reviewed the National Inpatient Database of lung resections and found that, overall, robotic resections had increased hospital costs. However, there was no analysis based on hospital volume of robotic cases [50]. In an analysis of the Nationwide Readmissions Database, Verma et al. stratified cost and outcomes by hospital volume. They found that high-volume hospitals had lower overall costs when performing robotic lobectomy [80]. This may reflect the impact of more experienced teams with streamlined operating systems. A recent RCT (RAVAL) comparing RATS to VATS lobectomies found that the incremental cost-effectiveness ratio (ICER) for robotic surgery was within the favorable limits despite increased costs compared to VATS. Given this, the authors concluded that robotic-assisted lobectomy was cost-effective compared to VATS [81]. 

A major contributor to the cost of robotic-assisted surgery is the operative time and maintenance. While the operative time does not always reflect the quality of the operation or post-surgical outcomes, it is a metric that impacts the total case number, patients treated, and hospital cost. As the volume and RATS experience of centers increase, operative time decreases, enabling the treatment of more patients. Lastly, newer robotic-system-acquisition models, such as leasing or “pay-per-use” contracts, have significantly changed the cost algorithms. 

## 7. Segmentectomy and Complex Pulmonary Resections

The standard of care for early NSCLC has historically been lobectomy [7]. Recently, two RCTs have compared sublobar resection to lobectomy for early NSCLC. In JCOG0802, 1106 patients were randomized to undergo a lobectomy or segmentectomy for Stage IA disease. Both the five-year overall survival and relapse-free survival of the segmentectomy group were noninferior to the lobectomy group. Despite similar survival rates, recurrence rates in the segmentectomy group were higher. The segmentectomy group had significantly improved FEV1 at the 1-year follow-up, despite not reaching the minimum threshold for clinical significance [82]. CALGB 140503 randomized 697 patients with Stage IA NSCLC to undergo either a lobectomy or sublobar resection. Sublobar resection, including wedge resection and segmentectomy, was noninferior to lobectomy for overall survival and disease-free survival [83].

With the acceptance of segmentectomy for lung cancer and the adoption of minimally invasive surgery, the outcomes for minimally invasive segmentectomy have significantly improved [84]. Thoracoscopic segmentectomy has similar nodal evaluation, postoperative complication rates, recurrence rates, and survival to thoracoscopic lobectomy [85,86,87,88]. Compared to open segmentectomy, the VATS approach has shown equivalent lymphadenectomy, decreased pulmonary complications, and improved disease-free and overall survival [89]. Robotic segmentectomy has been shown to be a safe operation with appropriate oncologic resection and survival [87,88,90,91,92,93]. In a review of early-stage NSCLC patients from the NCDB, patients who underwent minimally invasive segmentectomy had a shorter length of stay and similar nodal upstaging and long-term survival when compared to open segmentectomy [94]. Watkins et al. performed an analysis of the STS-GSTD from 2013 to 2021 of elective segmentectomies for NSCLC. Segmentectomy volume significantly impacted the choice of surgical approach (RATS, VATS, or open). High-volume centers (>16 cases per year) utilized RATS significantly more than medium- (4–16 cases per year) or low-volume (<4 cases per year) centers. RATS had greater nodal sampling, but N1 upstaging was more common in the open group. RATS had a significantly lower rate of conversion to open compared to VATS. MIS approaches had fewer postoperative complications and a significantly shower hospital stay compared to open [95]. 

Minimally invasive techniques have been established to be safe in complex pulmonary resections for lung cancer [96,97,98,99,100]. The improvements in patient outcomes after minimally invasive surgery are also seen in complex pulmonary resections. A review of the NCDB found that patients who underwent pneumonectomies via MIS had similar complication rates, perioperative mortality rates, and improved lymphadenectomy when compared to the open group [101]. In a comparison of open and MIS sleeve lobectomies, patients in the robotic group had a shorter operative time, similar complication rates, and improved survival rates compared to the open group. There was no difference in disease-free survival among the three techniques [102]. The patient outcome advantages of the VATS and RATS approaches are seen across simple lobectomies and segmentectomies to more complex pulmonary resections. 

## 8. Advancements in VATS and RATS

Since the introduction of VATS over thirty years ago, the idea of uniportal VATS (U-VATS) has surfaced. Rather than the traditional three- or four-port technique, uniportal surgery consists of a single incision, typically 4 cm or less [103]. The theoretical benefit of the uniport is the parallel instrumentation with the camera [104]. In a retrospective analysis of uniportal and multiportal VATS in North America, U-VATS was found to have a significantly shorter operative time, less blood loss, shorter chest tube duration, and a shorter length of stay. The rates of conversion to thoracotomy were not statistically different. Furthermore, there was no difference in the number of stations dissected, nodal upstaging, or margin positivity. The postoperative complications were also comparable [105]. Similar findings of shorter chest tube drainage and hospital stay were also seen in a systematic review comparing U-VATS and multiportal VATS [106]. Uniportal robotic-assisted lung resection is also feasible and safe, but it has been less studied [107,108,109]. In a smaller multi-center study, Manolache et al. compared uniportal RATS to multiport RATS. They found that uniportal RATS was used to perform significantly more segmentectomies. There was no conversion to open in the uniportal group. The operative time and length of stay were lower for uniportal RATS. There was no difference in 30-day mortality [108]. We anticipate significant growth in robotic uniportal thoracic surgery in the near future with the impending FDA approval of the robotic single-port system (SP, Intuitive Surgical, Sunnyvale, CA, USA).

One of the disadvantages of VATS is the lack of 3D visualization of the tissue. There is a loss of depth, thus making it harder to distinguish the proper planes. 3D technology has been introduced for VATS. Operating staff wear 3D glasses and use a specific 3D camera for VATS. Bagan et al. compared this technology with traditional 2D VATS. They found that blood loss, morbidity, duration of chest tube, and lymph node dissection were comparable. The operating time was significantly reduced in the 3D VATS cohort [110]. While 3D VATS has been shown to be feasible, there remain limited data on its use. 

For the identification of vasculature, planes, and tumors, the da Vinci robotic systems (Intuitive Surgical, Sunnyvale, CA, USA) have an intraoperative near-infrared fluorescence (Firefly) that can be used with indocyanine green (ICG). ICG can help identify aberrant vasculature, intersegmental planes, and small or deep tumors [111]. Pardolesi et al. demonstrated the delineation of intersegmental planes following ICG peripheral injection [112]. ICG can also be injected into specific lesions for identification. Geraci et al. described a method for using bronchoscopy preoperatively to identify and inject ICG into the target lesion. Peripheral ICG was also used to identify the planes. Using the Firefly, 86% of the 93 nodules were identified intraoperatively, and all patients had an R0 resection [113]. 

Another innovative method for the identification of nodules, particularly those with ground-glass opacities, is a fluorescent tracer (OTL38). OTL38 is a folate-receptor targeted fluorescent tracer that accumulates in malignant tissue and is visualized at specific wavelengths. It has been previously studied in VATS surgery with the use of a specific thoracoscope designed to detect the tracer (VATS-IMI) [114]. A “Sensitive Firefly” vision mode was created for the da Vinci platform in a pilot study of patients who received OTL38. The vision mode was specifically designed to identify tumor-specific fluorescence. The VATS-IMI thoracoscope was used afterwards to serve as a control for the “Sensitive Firefly” mode. The standard robotic white-light scope was able to identify seven out of ten of the pleural nodules based on standard signs of pleural puckering or distortion. The “Sensitive Firefly” and VATS-IMI scope were able to identify all ten of the nodules. There was no significant difference in the mean fluorescence intensity of the tumor, the mean fluorescence intensity of the background parenchyma, or the mean signal-to-background ratio between the “Sensitive Firefly” and VATS-IMI [115]. This is a powerful tool for surgeons, especially with the lack of haptic feedback of the robotic platform, to remove smaller and deeper nodules. 

One of the challenges in segmental lung resection is the variation in vascular anatomy and the identification of intersegmental planes. The concept of 3D-CT technology for preoperative planning has gained more traction [116,117,118]. Ujiie et al. have described the use of a preoperative planning system to map out 3D lung anatomy prior to surgical resection. This 3D model was integrated into the robotic platform for the surgeon to view via the console during the case to allow for identification of aberrant vascular anatomy [119].

Innovations in surgical technology can assist in preoperative planning and improve the precision and outcomes of minimally invasive surgery. 

## 9. Conclusions

The development of minimally invasive surgery has greatly advanced lung cancer treatment. VATS and RATS lung resection have consistently demonstrated enhanced patient outcomes compared to open lung resection via thoracotomy. Patients treated with minimally invasive techniques have fewer postoperative complications, improved pulmonary recovery, shorter hospital stays, less pain, and improved quality of life. Additionally, MIS for lung cancer has comparable oncologic outcomes related to overall survival, disease-free survival, and quality of lymphadenectomy. These improvements have led to a shift in standard-of-care treatment of lung cancer. With the rise of uniportal surgery, patient recovery may be even faster without compromising other outcomes. Advanced imaging techniques can offer improved identification of lesions or aberrant vasculature and allow for more tissue-sparing resections.

As minimally invasive surgery technology continues to evolve, the treatment of lung cancer will also evolve towards less invasive, safer, and more effective lung cancer operations. 

## Figures and Tables

**Table 1:** Outcomes of centers with low, medium, and high volumes of VATS or RATS lobectomy. ([2022] Servais et al. Reproduced with permission from authors [55]).

	Participant Center Volume Tertile			
	Low	Medium	High	*p*
VATS	n = 2092	n = 6569	n = 19,034	
Mortality, %	1.15	0.84	0.86	0.38
Major complication, %	7.90	7.19	6.25	0.002
Conversion to open, %	27.33	10.56	7.23	<0.001
RATS	n = 5683	n = 5599	n = 9107	
Mortality, %	0.83	0.80	0.96	0.56
Major complication, %	7.40	7.13	6.42	<0.001
Conversion to open, %	9.72	9.48	7.02	<0.001

RATS, robotic-assisted thoracoscopic surgery; VATS, video-assisted thoracoscopic surgery.
